# The mother – child nexus. Knowledge and valuation of wild food plants in Wayanad, Western Ghats, India

**DOI:** 10.1186/1746-4269-2-39

**Published:** 2006-09-12

**Authors:** Gisella Susana Cruz García

**Affiliations:** 1 Social Sciences Department, Wageningen University and Research Centre, Wageningen, The Netherlands; 2 Geverikerstraat 56, 6191 RP Beek (L), The Netherlands

## Abstract

This study focuses on the mother-child nexus (or process of enculturation) with respect to knowledge and valuation of wild food plants in a context where accelerated processes of modernization and acculturation are leading to the erosion of knowledge and cultural values associated with wild food plant use, in Wayanad, Western Ghats, India. Wild food plants in this biodiversity hotspot form an important part of local diets and are used as famine foods and medicines. In general, the collection and consumption of these foods are increasingly stigmatized as symbols of poverty and 'tribalness' (equivalent to 'backwardness'). The study, which falls within the discipline of ethnobotany, involves three socio-cultural groups – the Paniya and Kuruma tribes and non-tribals. Further, it examines the impact in the enculturation process of an unusual educational programme sponsored by the MS Swaminathan Research Foundation that is oriented towards creating awareness among children of cultural identity and local biological resources – the study compares children having participated in the programme with those who have not, with their mothers. The process of enculturation is assessed by comparing wild food plant knowledge and values between mothers and their children, and by examining events where knowledge transmission occurs, including collection and consumption. For that, quantitative and qualitative data collection and analysis tools were used, and methods included semi-structured interviews, photo identification and informal interviews of key informants.

Results ratify that women are the knowledge holders and are the primary means of knowledge transmission to their children. Nevertheless, fewer children are collecting wild food plants with mothers and learning about them, apparently because of children's lack of time. On the other hand, older people acknowledge that a "change in taste" is occurring among younger generations. In general, there is a simultaneous transmission from mothers to children of contrasting values pertaining to wild food plants: that they are 'good food' but also that they are symbols of low status and poverty, leading to feelings of shame and inferiority.

Finally, the study concludes that the educational programme, through a "learning by doing" approach counteracts social stigma and encourages learning among children of all ages and socio-cultural groups, particularly stimulating non-tribal children to learn from tribals.

## Background

Wayanad District, in the Western Ghats of India, is an area with great biological diversity where the tribal groups and other poor non-tribal peoples greatly depend on wild food resources for their subsistence – particularly on wild food plants (hereafter referred to as WFPs). WFPs are also renowned as famine foods in periods of food stress or shortage. WFPs, which are also important to cultural identity, provide essential sources of nutrients and vitamin A, as well as much seasonal dietary variety [1-3].

Nevertheless, it was reported in a recent unpublished study called 'Gender dimensions of wild food management in Wayanad, Kerala' (Narayanan MKR, Swapna MP and Kumar NA, 2004) made by the Community Agrobiodiversity Centre (hereafter referred to as CAbC) of the M.S. Swaminathan Research Foundation, that there is a decrease in both knowledge and consumption of WFPs mainly due to eroding availability of these plant resources and changing values around WFP consumption, which is increasingly seen as a symbol of poverty and 'tribalness'. The problem of erosion of cultural values and dietary traditions around WFPs due to social stigma has been reported for many other parts of the world. For example, in Tlaxcala, Mexico "social status restricts the use of a group of foods that could supplement an otherwise limited diet" [4], whereas in Swaziland the dependency on food imports and exotic vegetables has eroded WFP consumption and has "serious repercussions for food security and nutrition" [1]. While the previous study in Wayanad documented the range and diversity of WFPs consumed by the different socio-cultural groups in the area and noted with alarm the erosion of biological diversity and indigenous values, it did not investigate the latter issue in any depth.

Discrimination against tribal peoples is widespread, as is tribals' resentment of non-tribals: for example, regarding the Paniya tribe, "The general opinion of the non-tribal cultivators about the Paniyar is that they are undependable, slow and lazy. Whereas the Paniyar felt that the settlers were harsh, unkind and more demanding" [5]. There is a clash of cultural values: "The Paniyar, although they participate in the modern market economy, still hold a world view similar to their food gathering days" [5].

Schooling is a major problem for tribal children in Wayanad because, in spite of substantial efforts to promote it, they are not actively involved [6]. A major constraint is that different tribal groups have their own dialects, so language is a barrier [7,8]. The conventional structure of the educational system is not relevant for tribal children's lives in India [9]. Wayanad represents no exception to what has been widely reported across the globe – formal educational programmes very often have negative influences on indigenous values and practices (see e.g. the case for the Pacific Northwest of North America [10] and for Venezuelan Amazonia [11]).

The Community Agrobiodiversity Centre of M.S. Swaminathan Research Foundation was aware of the threats to local knowledge and cultural identity and its relations with welfare and biodiversity management in the area, and introduced an educational programme (hereafter referred to as EP) entitled "Every Child a Scientist" in Wayanad in 2001. This programme was intended to foster scientific inquiry among tribal and non-tribal children, providing an alternative to normal school curricula in the area. The objectives of the EP are "to promote knowledge around biodiversity heritage and create awareness among tribal and rural youth, improve the quality of student educational services using a multi-media training programme, explore and document bio-resources and their value to the community, generate a scientific temper and foster a 'Youth for Bioresources Conservation and Development' movement, identify pathways to conserve medicinal plants and herbal resources" [12].

The first question that arose on arrival in the research area was, "How do these children learn about WFPs and where do their values – both positive and negative – come from? There are several possible and probable answers: knowledge and values related to WFPs come from home, from schools, from the media and, in the case of Wayanad, from other educational efforts such as the Educational Programme "Every Child a Scientist". The transmission of knowledge and values – constituted by these and other factors and events – is complex and their various sources and influences are interrelated.

The objective of the study was to assess the process of enculturation – being defined as "the process by which human infants learn their culture" [13] – as evidenced by mother-child sharing of knowledge and values about WFPs and the influence of the EP in this process. The previous research determined that it is primarily women who are involved in WFP collection and use, and women are the main knowledge holders with respect to WFPs and are responsible for collection, processing and preparation of these plants for home consumption. The assumptions were that mothers transmit knowledge and values about WFPs to their children and that the practices of collection and consumption of WFPs are prime instances in which social learning takes place.

## Methods

### Study area

Wayanad district, situated in the Western Ghats in the north-eastern part of the state of Kerala, India, is considered one of the world's biodiversity hotspots, since it hosts great richness of flora and fauna [14]. It has an area of 2136 km^2^, where 37% of the land area is forest covered and 55% cultivated by agriculture. The tribal population represents 17% of the total population of the district, and is the largest tribal population in the state of Kerala [6]. The district is characterised by high ethnic diversity, with five dominant tribal groups – Kurichiya, Kuruma, Paniya, Adiya and Kattunaikka – and seven minor communities [14]. The present study includes three socio-cultural groups: two tribes (Paniya and Kuruma), and non-tribal rural communities.

The Paniya, a landless tribe, constitute 46 % of the total tribal people in Wayanad, and are the largest scheduled tribe in Kerala. Most of them depend upon wage labour in the paddy fields and on farms of the landowning classes. They consume the highest quantity of WFPs (152 species) in relation to the other socio-cultural groups that have been studied in the area, and are considered "famed wild leaf eaters" (Narayanan MKR, Swapna MP and Kumar NA, 2004, unpublished). The Kuruma are a landowning tribe that constitute 14.6% of the tribal population in Wayanad. In comparison to tribals, non-tribals are better-off economically and have more capacity to purchase food in markets, and also maintain home gardens.

### Data collection

Fieldwork was conducted between July and October 2004 with a research population of 81 children, 22 teens, 57 mothers, and key informants. Initially the teens (from 15 to 18 years old) were considered within the group of children, but later they were excluded because, from a local point of view, teens above 15 years of age are not considered 'children'. Four main variables were considered for children and teens stratification: participation in the EP, socio-cultural group (Paniya, Kuruma, non-tribal), age and sex.

All Paniya, Kuruma and non-tribal children and teens that participated in the EP were included in the study (n = 33), and were interviewed when they came to the CAbC to follow the normal programme. Those children and teens that did not participate in the EP (n = 70) were selected through convenience sampling attempting to have a balanced number of informants according to the stratification variables. Researchers visited tribal colonies and non-tribal rural villages and invited adults to take their children to the CAbC in the scheduled weekends, when games, group discussions, lunches, and the individual interviews were organized. Children were not interviewed at home because it was not possible to obtain an individual child interview without the intervention of the mother. Children were not interviewed at school in order to also include in the sample children that dropped out or did not attend frequently.

The tribal colonies of Puthoorvayal Paniya, Mutharikunu Paniya, Mangavayal Paniya, Rattakolly Paniya, Chandempety Kuruma, Mangavayal Kuruma, and Puthoorvayal Kuruma, and the non-tribal rural villages of Mandakakuni, Meppadi, Chooralmala and Puthoorvayal were visited. In general, 26 Paniya, 34 Kuruma and 43 non-tribal children and teens were interviewed.

The 57 mothers of some of the interviewed children (24 whose children participated in the EP, 33 whose children did not) were selected by convenience sampling: those who were present and willing to collaborate when researchers visited their colonies or villages for a second time, a few weeks after the interviews of their children had finished. Mothers were interviewed at home.

Both qualitative and quantitative data gathering and analysis tools were used. Research instruments included semi-structured interviews, photo identification of selected WFPs, and informal key informant interviews. All semi- and fully structured data collection instruments were pre-tested with informants that were not later represented in the sample, in order to determine whether the questions generated the desired information [15].

Semi-structured interviews were very similar for mothers and children. They were employed firstly to reveal general information about collection and consumption of WFPs, such as frequency of consumption and with whom they go for collection; secondly to assess their perceptions of their own and other's social attitudes towards collection and consumption; thirdly to investigate children's and mother's perceptions of each other's knowledge; fourthly to compare their value categories relating to WFP importance.

The photo identification of WFPs investigated the ability of informants to identify 26 selected WFPs – verified as consumed by the three socio-cultural groups – through photographs, providing a 'correct' vernacular name for the plants that informants could identify (secondary materials were used for identification of species names). The 26 WFP photos – showing the edible part (leaf, tuber and/or fruit) – were numbered and given to each informant individually. They were then asked to indicate the number of the photo and the vernacular name of the plant. Tribal mothers were unable to identify plants through photos, so this exercise could only compare non-tribal children with their non-tribal mothers. The results were analysed using SPSS 12.0.1, and Spearman's rho correlations were carried out in order to compare the results.

Finally the informal interviews of the key informants were held with experienced elderly people from the selected colonies and rural villages with the main objective to assess a perceived change in WFP consumption.

## Results and discussion

### Practices of collection and consumption as a learning process

Collection and consumption of WFPs were assessed as learning events where knowledge is transmitted. Of course children not only learn about WFPs by collecting them together with people from whom they learn, but also by preparing and consuming food made from WFPs. However, with respect to the preparation of WFPs, during the pre-tests of the interviews, boys said they did not participate whereas girls did, so this is not a satisfactory way to determine from whom boys learn about WFPs, at least as a practice.

All mothers collect WFPs – providing WFPs for their households. It is remarkable that the most common channel of social learning is through the mother, or female relatives in general, which was reflected in the fact that most children (71%) collect with them. Nevertheless this process of learning is being weakened, which is observed in the fact that almost half of the mothers (44%) said that they collect WFPs alone. Their explanation is that children have a lack of time, or that WFPs are less available – reasons also considered by children. Even more, with respect to non-tribal people, those children that did not participate in the EP could identify far less WFPs than their mothers did. This is alarming, since there is also an increasing process of WFP stigmatization.

Other important channels of knowledge transmission are friends (41% of children) and neighbours, especially in the case of tribal children who collect in groups. An important instance for learning is collecting for consumption on the spot, which mostly occurs on the way to school – in this activity children have the opportunity to share their knowledge with friends. Nevertheless, this practice, which has decreased with age and through lack of time, is less common among older children who take buses to school.

With respect to WFP consumption, all mothers and children consume WFPs, but tribal people consume WFPs more frequently than non-tribals do. What is surprising is the case of several Paniya children that did not participate in the EP: they said they do not consume WPFs more than once a week, but their mothers said they do; the children who participated did not deny that.

### Comparing non-tribal mothers' and their children's wild food plant knowledge

Non-tribal mothers' and their children's knowledge of WFPs are highly correlated (Spearman's rho correlation coefficient is significant at the 0.01 level), because the WFPs that mothers identify are related to those that children identify. The WFPs that were identified by 75% or more of the non-tribal mothers (n = 25) were: *Amaranthus spinosus *L.("*mullancheera"*), *Cassia tora *L. ("*thavara"*), *Alternanthera sessilis *(L.) DC. ("*ponnamkanny"*), *Colocasia esculenta *Schott ("*thalu"*), *Centella asiatica *Urban ("*muthilila"*), *Diplazium esculentum *(Retz.) Sw ("*churuli"*), *Bacopa monnieri *(L.) Pennell ("*brahmichappu"*) and *Solanum nigrum *L. ("*mudanga"*). These were also among the most identified WFPs by non-tribal children (n = 19) and teens (n = 8), with the exception of *Centella asiatica *Urban that was identified by all children that participated in the EP but only by 14% of those children that did not. On the other hand, *Amaranthus viridis *L. was identified in a greater number by children and teens than by mothers (see Table 1).

**Table 1:** Non-tribal children's, teens' and mothers' wild food plant identification according to participation in the Educational Programme.

Wild food plants*	**Mothers**		Children**				**Teens**	
	
			**Did not participate EP**		**Participated EP**		**Participated EP**	
	
	n = 25		n = 14		n = 5		n = 8	
	
	count	%	count	%	count	%	count	%
*Alternanthera sessilis *(L.) DC.	23	92	7	50	5	100	8	100
*Amaranthus spinosus *L.	24	96	11	79	5	100	7	88
*Amaranthus viridis *L.	12	48	13	93	4	80	7	88
*Arenga wightii *Griff.	3	12	1	7	2	40	4	50
*Bacopa monnieri *(L.) Pennell	19	76	8	57	5	100	8	100
*Bidens biternata *Merr. & Sherff	11	44	1	7	4	80	7	88
*Cassia tora *L.	24	96	9	64	5	100	8	100
*Centella asiatica *Urban	20	80	2	14	5	100	7	88
*Colocasia esculenta *Schott	23	92	14	100	4	80	6	75
*Cyathula prostrata *Blume	9	36	1	7	2	40	5	63
*Cycas circinalis *Roxb	5	20	0	0	3	60	3	38
*Dioscorea belophylla *Voigt	3	12	1	7	1	20	3	38
*Dioscorea kalkapershadii *Prain & Burkill	8	32	2	14	1	20	3	38
*Dioscorea oppositifolia *L.	6	24	0	0	4	80	6	75
*Dioscorea wightii *Hook.f.	1	4	1	7	0	0	2	25
*Diplazium esculentum *(Retz.) Sw	20	80	10	71	5	100	8	100
*Hygrophila erecta *(Burm.f.) Hochr.	0	0	0	0	1	20	2	25
*Lycianthes laevis *Dunal	0	0	1	7	2	40	2	25
*Momordica subangulata *Blume	8	32	2	14	4	80	7	88
*Mukia maderaspatana *M.Roem.	4	16	1	7	2	40	1	13
*Passiflora calcarata *Mast.	9	36	0	0	2	40	1	13
*Physalis minima *L.	6	24	0	0	0	0	1	13
*Portulaca oleracea *L.	4	16	0	0	1	20	2	25
*Solanum nigrum *L.	19	76	8	57	2	40	4	50
*Trianthema portulacastrum *L.	16	64	0	0	2	40	4	50
*Waltheria indica *L.	4	16	0	0	2	40	2	25

* Latin plant names quoted according to the Index Kewensis (IK), as referred in The International Plant Names Index (IPNI) [17].

** 13 non-tribal children did not show

Non-tribal children who participated in the EP (mean = 17.4) could identify more WFPs than non-tribal mothers (mean = 11.2). Non-tribal children learned about them through EP curriculum or because of their exposure to tribal children's knowledge. Both mothers and children are totally aware of that. On the other hand non-tribal mothers could identify more plants than non-tribal children who did not participate in the EP (mean = 6.6), not only because children are still engaged in a learning process, but also because there is apparently a process of cultural erosion which mothers and children mainly blame on school attendance, on lack of interest on their children's part, and on decreasing availability of WFPs. Compared to the teens the latter could identify more WFPs than the other groups (mean = 19.9).

### Comparing mothers' and children's wild food plant cultural valuation

Most criteria for valuating WFPs according to importance were transmitted from mother to child. Children learned from their mothers that WFPs are "healthy", "do not contain chemicals", and are "medicinal". On the other hand it is clear that the criteria "taste" and "fresh" are clearly children's own. Predictably children are more motivated by factors such as taste and texture.

There is criteria transmission between mothers and children in terms of perceptions of others' social attitudes towards WFP collection and consumption, and in special social stigma. Both mothers and their children who have feelings of shame with respect to WFPs, gave similar explanations for the stigmatization of those practices. For instance both explained that they dislike other people seeing them collecting WFPs for reasons of "shame" and "status". Nevertheless whereas children have more social stigma concerning collection than consumption of WFPs, mothers show more social stigma related to consumption.

All mothers consent to the fact that they used to consume more WFPs before, and report decreasing WFP collection due to decreasing availability of WFPs rather than to increasing social stigma. On the other hand experienced elderly people acknowledge that a "change in taste" is occurring among younger generations due to more interactions with other social groups. The idea of "change of taste" could be related to the introduction of different "modern" food items that are consumed by people from social groups with higher income who mostly come from towns and cities. This could be reinforced by mothers disliking to offer WFPs to their guests (52% of mothers) and to prepare WFPs in festivities (63%) due to "status" and "taste preference".

## Conclusion

Practices of collection and consumption of WFPs are prime instances in which social transmission of knowledge and values with regard to WFPs takes place. Findings have demonstrated that this process is influenced by participation in the EP and the interaction with other socio-cultural groups (tribal – non-tribals). Social stigma is to some extent transmitted by mothers. There is a simultaneous transmission from mothers to children of dual positive-negative valuation criteria: they are aware that WFPs are "good food" and "healthy" but they also relate them to "low status" and "poverty", which leads to feelings of shame and inferiority.

The findings reinforce the conclusions reported in previous research that there is an alarming erosion of traditional botanical knowledge. In this study, this appears with respect to the following five aspects:

     • All mothers collect WFPs, whereas not all children do.

     • Almost half of the mothers said that they collect WFPs alone, whereas collection used to be a knowledge-transmitting event.

     • All mothers consent to the fact that they consumed more WFPs in the past.

     • There is a growing process of social stigmatization related to WFPs, which leads to a lack of interest in WFP knowledge acquisition in children.

     • With respect to non-tribal people the children that did not participate in the EP could identify far fewer WFPs than their mothers.

This knowledge erosion is explained by mothers and children mainly because of less WFPs being available, and through children's lack of time due to school attendance. This is quite important since formal education does not consider local resources, knowledge and culture; the conventional structure of the educational system is not relevant in this regard to the lives of tribal children. On the other hand elderly people explained the change in consumption of WFPs as an ongoing process of "change of tastes" in younger generations towards market foods as new status symbols. The acquisition of new tastes, food attitudes and the adoption of new status symbols have been reported by several authors across many parts of the world. Lewis called it "gustatory subversion" [16], which refers to the introduction of exotic foods that have the effect of subverting local cuisine, resulting in a low quality diet and economic dependence.

It has been observed that the EP "Every Child a Scientist" influences children's knowledge and valuation of WFPs, their attitudes and self-confidence, which is an explicit goal of the EP. The "learning by doing" approach and the emphasis on "breaking the ice" and promoting the interaction between girls and boys, tribals and non-tribals, and among younger and older children is innovative and unusual for this caste society, which stimulates children to learn from and respect each other.

## List of abbreviations used

WFP Wild Food Plant

CAbC Community Agrobiodiversity Centre of the M.S. Swaminathan Research Foundation

EP "Every Child a Scientist" Educational Programme
